# Linking Electronic Health Records for Multiple Sclerosis Research: Comparative Study of Deterministic, Probabilistic, and Machine Learning Linkage Methods

**DOI:** 10.2196/79869

**Published:** 2026-02-04

**Authors:** Ohoud Almadani, Yasser Albogami, Adel Alrwisan

**Affiliations:** 1 Saudi Food and Drug Authority, 4904 northern ring branch rd, 13513, Hittin Dist, Unit number : 1, Riyadh, 13513, Saudi Arabia, 966 555300732; 2 Department of Clinical Pharmacy, College of Pharmacy, King Saud University, Riyadh, Saudi Arabia

**Keywords:** EHR, MS, probabilistic, deterministic, ML, multiple sclerosis, electronic health record, machine learning

## Abstract

**Background:**

Data linkage in pharmacoepidemiological research is commonly employed to ascertain exposures and outcomes or to obtain additional information on confounding variables. However, to protect patient confidentiality, unique patient identifiers are not provided, which makes data linkage across multiple sources challenging. The Saudi Real-World Evidence Network (SRWEN) aggregates electronic health records from various hospitals, which may require robust linkage techniques.

**Objective:**

We aimed to evaluate and compare the performance of deterministic, probabilistic, and machine learning (ML) approaches for linking deidentified data of patients with multiple sclerosis (MS) from the SRWEN and Ministry of National Guard Health Affairs electronic health record systems.

**Methods:**

A simulation-based validation framework was applied before linking real-world data sources. Deterministic linkage was based on predefined rules, whereas probabilistic linkage was based on a similarity score–based matching. For ML, both similarity score–based and classification approaches were applied using neural networks, logistic regression, and random forest models. The performance of each approach was assessed using confusion matrices, focusing on sensitivity, positive predictive value, *F*_1_ score, and computational efficiency.

**Results:**

The study included linked data of 2247 patients with MS from 2016 to 2023. The deterministic approach resulted in an average *F*_1_ score of 97.2% in the simulation and demonstrated varying match rates in real-world linkage: 1046/2247 (46.6%) to 1946/2247 (86.6%). This linkage was computationally efficient, with run times of <1 second per rule. The probabilistic approach provided an average *F*_1_ score of 93.9% in the simulation, with real-world match rates ranging from 1472/2247 (65.5%) to 2144/2247 (95.4%) and processing times ranging from approximately 0.1 to 5 seconds per rule. ML approaches achieved high performance (*F*_1_ score reached 99.8%) but were computationally expensive. Processing times ranged from approximately 13 to 16,936 seconds for the classification-based approaches and from approximately 13 to 7467 seconds for the similarity score–based approaches. Real-world match rates from ML models were highly variable depending on the method used; the similarity score–based approach identified 789/2247 (35.1%) matched pairs, whereas the classification-based approach identified 2014/2247 (89.6%).

**Conclusions:**

Probabilistic linkage offers high linkage capacity by recovering matches missed by deterministic methods and proved to be both flexible and efficient, particularly in real-world scenarios where unique identifiers are lacking. This method achieved a great balance between recall and precision, enabling better integration of various data sources that could be useful in MS research.

## Introduction

Real-world data (RWD) derived from routine clinical care provide remarkable potential for generating pharmacoepidemiological studies related to medication safety, effectiveness, and utilization [1-4]. The recent emergence of electronic health records (EHRs) in the Kingdom of Saudi Arabia led to the foundation of the Saudi Real-world Evidence Network (SRWEN) by the Saudi Food and Drug Authority [5-8]. This central network plans to integrate and harmonize EHRs from various hospitals, primarily to maximize the efficacy of RWD in contributing to drug regulatory policymaking and understanding disease dynamics in the Saudi population [6,7].

Common data models (CDMs), such as the observational medical outcomes partnership model, incorporate consolidated formats necessary for data standardization across multiple clinical data sources to perform large-scale observational studies [9-11]. CDMs enable researchers to pool multiple EHR datasets and perform federated data analyses through standardized data architectures, formats, and terminologies [10,11]. The observational medical outcomes partnership CDM, developed as part of the observational health data sciences and informatics, offers a systematic framework of clinical data across diverse subdomains such as demographics, diagnoses, medications, procedures, and laboratory results. It uses standardized terminologies, mitigating inherent variability, customized formats, or inconsistent coding in raw EHR data [9,11].

While CDMs are in place to standardize structures in collecting and evaluating clinical data, many are built on deidentified datasets that may not hold vital contextual information or complete patient histories [9-11]. Thus, restricting the ability to solve such limitations of CDM as incomplete data mapping, the lack of direct mappings for some clinical or medication concepts, laboratory or narrative data gaps, which necessitate going back to the data to retrieve context-specific information that may not be contained in the standardized model [9-11]. Therefore, EHR linkage offers a powerful solution addressing this limitation by enabling researchers to link patient records across various databases and reconstruct comprehensive longitudinal health trajectories [12-14]. Common linkage approaches are deterministic, probabilistic, and machine learning (ML) linkage, each with advantages and challenges [14-16]. For instance, the deterministic linkage is based on an exact matching across predefined variables, achieving high precision; however, sensitivity may be compromised in this case, particularly when matching data that contain errors in a given variable. Probabilistic linkage overcomes this weakness by using weighted matching, enhancing sensitivity at the cost of reduced precision in specific applications [12,13]. ML techniques, such as gradient boosting and neural networks (NNs), can achieve high levels of accuracy [12,13]. These models are particularly successful when integrating a dataset with large volumes of missing and errant data, identifying more links than deterministic and probabilistic approaches [16].

With a growing number of multiple sclerosis (MS) cases among the Saudi population—from 25 per 100,000 in 1998 to 40.4 per 100,000 in 2020—the linkage of data pertaining to MS is of priority, especially given the complexity of MS treatment management, both within Saudi Arabia and globally [17]. SRWEN contains a complex variety of data sources, reference standards, and case definitions, highlighting the intrinsic complexity associated with the reliability of RWD in health care settings [7]. Therefore, accurate linkage of EHRs is paramount for addressing the complexities associated with defining patient outcomes with MS, such as disease progression, relapse rate, and treatment response. This analysis requires frequent validation by clinical expertise and enhancement of the validity and robustness of the SRWEN[17]. To achieve this, we compared different EHR linkage methods, including deterministic, probabilistic, and ML approaches. The findings from this study may have implications for the value and design of future longitudinal studies using linked MS data.

## Methods

### Ethical Considerations

This study was approved by the institutional review board of Saudi Food and Drug Authority with approval number 2024_17 and by the King Abdullah International Medical Research Center with approval number ROMJ/WE/WS/059/2023. Because the data were extracted from the EHR system as part of routine clinical care, patients are required to provide consent to receive treatment within the institution. Separate study-specific consent was considered waived in accordance with institutional policies. Patient privacy and confidentiality were granted through de-identification of all records prior to data exchange, such that no direct identifiers were available to the research team.

### Study Population and Data Sources

In this study, we used 2 datasets from 2 different sources. First, we included SRWEN data of patients with MS between 2016 and 2023. The SRWEN functions as a repository of longitudinal health care information and incorporates diverse EHR data, such as demographics, medical history, medication use, laboratory outcomes, hospitalizations, and clinical outcomes. Information is gathered from diverse EHRs from multiple hospitals in Saudi Arabia, including the Ministry of National Guard Health Affairs (MNGHA). Subsequently, these collected data are standardized through a CDM to ensure consistency and streamline analysis [6]. Furthermore, an anonymization process is implemented prior to exchanging data to ensure patient privacy [6]. For the purposes of this study, we focused exclusively on extracting and analyzing MNGHA data within SRWEN.

Similarly, we collected MNGHA EHR data from King Abdullah International Medical Research Center, identifying patients with MS using the International Classification of Diseases 10th version (*ICD10*: G35) for the same period of (2016‐2023). MNGHA EHRs contain routinely collected data, including patient characteristics, diagnostic history, medications history, and visit records (ie, admissions, outpatient clinical visits, or emergency visits; Table S1 in Multimedia Appendix 1).

While SRWEN collects data already from the MNGHA, the benefit of the direct link between these 2 entities is advantageous. Data within SRWEN are organized and standardized within a CDM, providing for multi-institutional data analysis at the risk of some loss in source detail. By linking the primary MNGHA EHRs to their corresponding RERN representations, we were able to recover additional clinical granularity from narrative diagnosis notes and reports. This linkage also allowed validation of data consistency between SRWEN and its original source, resulting in a larger and more accurate longitudinal profile of patients with MS.

### Simulation-Based Validation

#### Overview

We first cloned and deidentified the original dataset to address the challenge of linking deidentified records from the targeted database (ie, SRWEN) with original records (ie, MNGHA EHRs). This process ensured a realistic framework testing and that the dataset resembled the deidentified target dataset. Simulating the conditions of the intended linkage objective, we linked records to themselves using these deidentified cloned records, employing deterministic, probabilistic, and ML approaches. Following this self-linkage phase, we assessed each approach’s performance using computational efficiency metrics. Consequently, the best-performing linkage method was used to link the original records to the target dataset, thereby maximizing the accuracy and reliability of the final linkage. This methodology enabled a rigorous, performance-based selection of the optimal linkage approach for secure and accurate record linkage of deidentified datasets [13].

#### Deterministic Approach

Deterministic EHR linkage is a rule-based approach that relies on exact matches between specific patient identifiers across different EHR systems [12,14,15,18]. Given the lack of patient identifiers in SRWEN, we implemented multiple matching criteria (ie, rules) based on a range of available variables, each considered both independently and in unique combinations (Table S2 in Multimedia Appendix 1). We explored different variable groupings in some detail to determine how various linkage methods perform under conditions with differing degrees of completeness and complexity of data encountered in real-world settings. This approach generates pairs of records classified as match or nonmatches. For each pair, we included multiple linkage variables, including demographic characteristics (eg, gender, date of birth, nationality, eligibility), and health care–related fields (eg, *ICD-10* codes, diagnosis description and date, medication order details, visit type and date, and facility information) [12,14,15,18]. Additional derived variables were generated from these data elements, including coverage, which was derived from eligibility and indicated a patient’s treatment eligibility, the first MS diagnosis date, derived from the diagnosis data, and inpatient admission status, derived from visit records.

#### Probabilistic Approach

In this approach, patient records were grouped into blocks based on shared variables (eg, date of birth, initial MS diagnosis date, received medications, or admission events) to reduce the number of pairwise comparisons required for linkage [12,14,19]. Subsequently, each record pair within a block was compared across diverse fields, including gender, coverage (ie, eligibility), region, nationality, facility name, or diagnostic codes other than MS [12,14,19]. A similarity score was assigned to each record pair corresponding to the level of agreement on predefined variables. Variable weights were estimated as match probabilities (ie, m-probability)—the probability that two field values match, given that the records refer to the same individual—and unmatch probability (ie, u-probability)—the probability that 2 field values match given that the records refer to different individuals. These probabilities were computed using the expectation-maximization algorithm [12-14,19]. An overall similarity score for each pair was computed by summing the weighted scores across all variables [12,14,19].

Pairs were categorized as matches or nonmatches using a one-to-one linkage approach. This method was implemented using the “select_n_to_m” function from the rclin package, which maximizes total similarity score under the constraint that each record is linked only once [20]. This probabilistic approach incorporates imperfect matches caused by inaccuracies or missing data, thereby enhancing the quality and reliability of linked EHR datasets in clinical studies [19].

#### ML Approach

In the ML approach, record linkage was performed in 2 methods. First, we generated pairs of records using common variables in the cloned data to limit the number of comparisons, creating a grouped set of pairs [20] Similarity scores were computed for each pair across different selected variables 20[]. Pairs were labeled as matches or nonmatches for training using the known true matches. The pairs were then fed into different models for training, and we fitted the model on variables that had the higher impact based on each variable similarity score, allowing the model to learn patterns that accurately identify true matches [20].

Second, a classification-based ML approach was implemented by training models on a labeled training dataset comprising 75% of all possible record pairs derived from the original dataset. Match and nonmatch labels were derived from the reference dataset. The remaining 25% of the record pairs were used for the testing dataset to evaluate the classifier performance [16].

Four ML algorithms—K-nearest neighbor, logistic regression (LR), NN, and random forest (RF)—were used in both methods to evaluate their linkage accuracy [16]. Internal validation was reinforced using a 100-replication bootstrap approach in each method for more robust performance estimates and to ensure stability across repeated sampling iterations [16]

### Real-World Record Linkage

In the real-world matching scenario, we linked data from SRWEN and MNGHA to identify patients diagnosed with MS between 2016 and 2023. Because the SRWEN and MNGHA datasets lack a unique common identifier, no reference standard was available to evaluate the performance of each matching algorithm [13]. Therefore, model performance was compared based on the number of matched record pairs identified.

### Statistical Analysis

Descriptive analyses of both datasets, including diseases, demographics, and facilities, were tabulated and presented by counts and percentages. Using original identifiers as the reference standard, accurate matches and true nonmatches were defined for the simulation [13]. True matches were record pairs agreeing on identifiers, whereas true nonmatches were record pairs conflicting on identifiers [13]. Sensitivity and positive predictive value (PPV) were calculated along with their corresponding 95% CI. Additionally, the *F*_1_ score was calculated as a measure of balanced accuracy between sensitivity and PPV (equation 1) [19]. Computational time required by the ML model to complete the record linkage process.


(1) $$F_{1}score=2\times \frac{Sensitivity\times PPV}{Sensitivity+PPV}$$

## Results

### Descriptive Analysis

The MNGHA dataset contained 2642 patients with MS, whereas 2247 were identified from SRWEN. Because only MNGHA-related dataset within SRWEN was used, 395/2642 (14.9%) patients with MS from the original MNGHA dataset were not captured in the SRWEN data. The distribution of demographic data between the 2 data sources was comparable. For instance, the mean age of patients in the MNGHA dataset was 34.4 (SD 11) years, which was similar to the mean age of 34.6 (SD 11.3) years in SRWEN.

### Deterministic Approach

The highest number of pairwise comparisons (3,854,740) was observed in the “sex” variable, compared with a significant precise pairwise comparisons (6212) using the “date of birth (DOB)” or “nationality” variables. Sensitivity was consistent with a perfect score across the variables. However, the specificity rate varied, with the highest (99.9%) observed using “DOB” and the lowest (5%) observed using “nationality.” Conversely, precision was considerably low across different variables, with the highest (42.5%) observed using “DOB.” Moreover, both “DOB” and “MS Date” had relatively high *F*_1_ scores of 59.7% and 48.8%, respectively (Table 1).

**Table 1.** Results of deterministic matching in % based on an individual variable.

Metric	Sex	DOB^a^	Nationality	Coverage	Facility	Region	MS^b^ date^c^	Admissions	Other diagnoses	Medications
Accuracy	44.45	99.95	5.09	69	62.92	58.06	99.92	88.15	64.37	99.73
Sensitivity (recall)	100	100	100	100	100	100	100	100	100	100
Specificity	44.43	99.95	5.05	68.99	62.91	58.04	99.92	88.14	64.36	99.73
PPV^d^ (precision)	0.07	42.53	0.04	0.12	0.10	0.09	32.28	0.32	0.11	12.45
*F*_1_ score	0.14	59.68	0.08	0.25	0.21	0.18	48.81	0.64	0.21	22.14

a DOB: date of birth.

b MS: multiple sclerosis.

c MS date: first multiple sclerosis diagnosis.

d PPV: positive predictive value.

A sequence of deterministic linkage rules was applied to evaluate data matching using a set of variables instead of a single variable (Table 1). The number of correctly matched pairs remained consistent across most rules, with 2642 pairs correctly matched for each rule. Likewise, almost all of the linkage rules resulted in a nearly perfect score across the metrics. The average *F*_1_ score for the deterministic approach was 97.2% (Table S3 in Multimedia Appendix 1). In terms of computing time, deterministic linkage was completed in less than one second for each criterion (Table S3 in Multimedia Appendix 1).

### Probabilistic Approach

To ensure a manageable dataset for analysis, we first reduced the number of comparisons by blocking records based on exact matches on DOB (6212 pairs). Precision remained consistently perfect regardless of the variables used across diverse combinations of matching criteria in the probabilistic approach. Both specificity and sensitivity increased with more variables, as the combination of sex, nationality, and coverage was 76%. Further, adding the facility name and the MS diagnosis date led to a perfect score. A similar observation was found for the *F*_1_ score, which began at 69.9% with sex only, increased to 94.6% after adding facility name and region, and reached a perfect score of 100% with the inclusion of MS diagnosis date.

By using a combination of the best-performing variables derived from the deterministic approach to improve match accuracy further, in addition to examining different blocking criteria, precision was constantly at 100%. Sensitivity and specificity rates were high, considerably assisted by combining specific clinical variables. Likewise, the *F*_1_ score ranged from 82% to 99.9% (Table S3 in Multimedia Appendix 1). In terms of computing time, probabilistic linkage required 0.1 to 5 seconds for each criterion (Table S3 in Multimedia Appendix 1).

Using this probabilistic approach with the one-to-one linkage (ie, select_n_to_m from the reclin package) matching criterion, the linkage models achieved perfect performance for all the metrics evaluated. In addition, m-probabilities and u-probabilities were compared across the variables under different matching criteria (Table S4 in Multimedia Appendix 1).

### ML Approach

In the similarity-score method, models trained on combinations of DOB, MS diagnosis date, and medications, after being blocked (ie, exact match) on admission, performed best overall. NN, RF, and LR models demonstrated comparable performance, with slightly higher accuracy using NN (99.37% vs 99.36%). However, computational time analysis showed that LR presented a combination of high performance and acceptable processing time (15‐766 s). In contrast, both RF and NN, while having comparable results to LR, were less practical for large-scale linkage tasks due to their longer training times (15‐7467 s) associated with their complexity (Table S5 in Multimedia Appendix 1).

In the classification approach, despite the blocking variable or combination of matching criteria, performance was constantly high among the three models, ranging from 91.7% to 100%, while computational efficiency varied depending on the blocking strategy and applied comparison criteria. Moreover, the classification is usually more computationally expensive (13‐16,936 s) compared with the similarity-score approach, affecting scalability for larger datasets. However, K-nearest neighbor demonstrated an excellent trade-off between computational efficiency and linkage performance (Table S5 in Multimedia Appendix 1).

### Record Linkage

The deterministic approach produced the fewest matched pairs. Increasing the number of variables (eg, adding admissions or medications) made the linkage criteria stricter, resulting in gradually fewer matched pairs, ranging from 1046/2247 (46.6%) to 1946/2247 (86.6%). The probabilistic approach achieved more pairs ranging from 1472/2247 (65.5%) to 2144/2247 (95.4%) based on the combination of variables. The ML approach, using similarity scoring or classification with an NN model, generated 789/2247 (35.1%) pairs and 2014/2247 (89.6%) pairs, respectively (Table S5 in Multimedia Appendix 1).

## Discussion

### Principal Results

Through the simulated scenario and after using a mixture of variables without identifiers, we determined that the deterministic method was the most efficient data linkage algorithm. Although this approach had limited generality, it accomplished 100% scores across all metrics without incurring high computational times. Therefore, the deterministic approach is practical when we need absolute certainty in matched pairs. In contrast, the probabilistic method also demonstrated efficient performance with a similar mixture of variables. Initially, the recall and specificity metrics exhibited low performances. However, when we applied one-to-one linkages, the performance of the probabilistic method improved significantly, yielding a 100% score. Furthermore, the core benefit of this approach is retrieving a higher number of matched pairs, signifying its advantage when maximum generalization and thorough data retrieval are required. In the case of ML methods like NN models, we attained perfect performance scores at the expense of higher computational costs, limiting their feasibility in real-world scenarios. Among all methods, the probabilistic approach provided the highest number of patients, signifying its flexibility in real-world healthcare scenarios. These outcomes give a reproducible framework for appropriate EHR record linkages, underscoring its efficacy in practical patient care situations.

**Figure 1.**
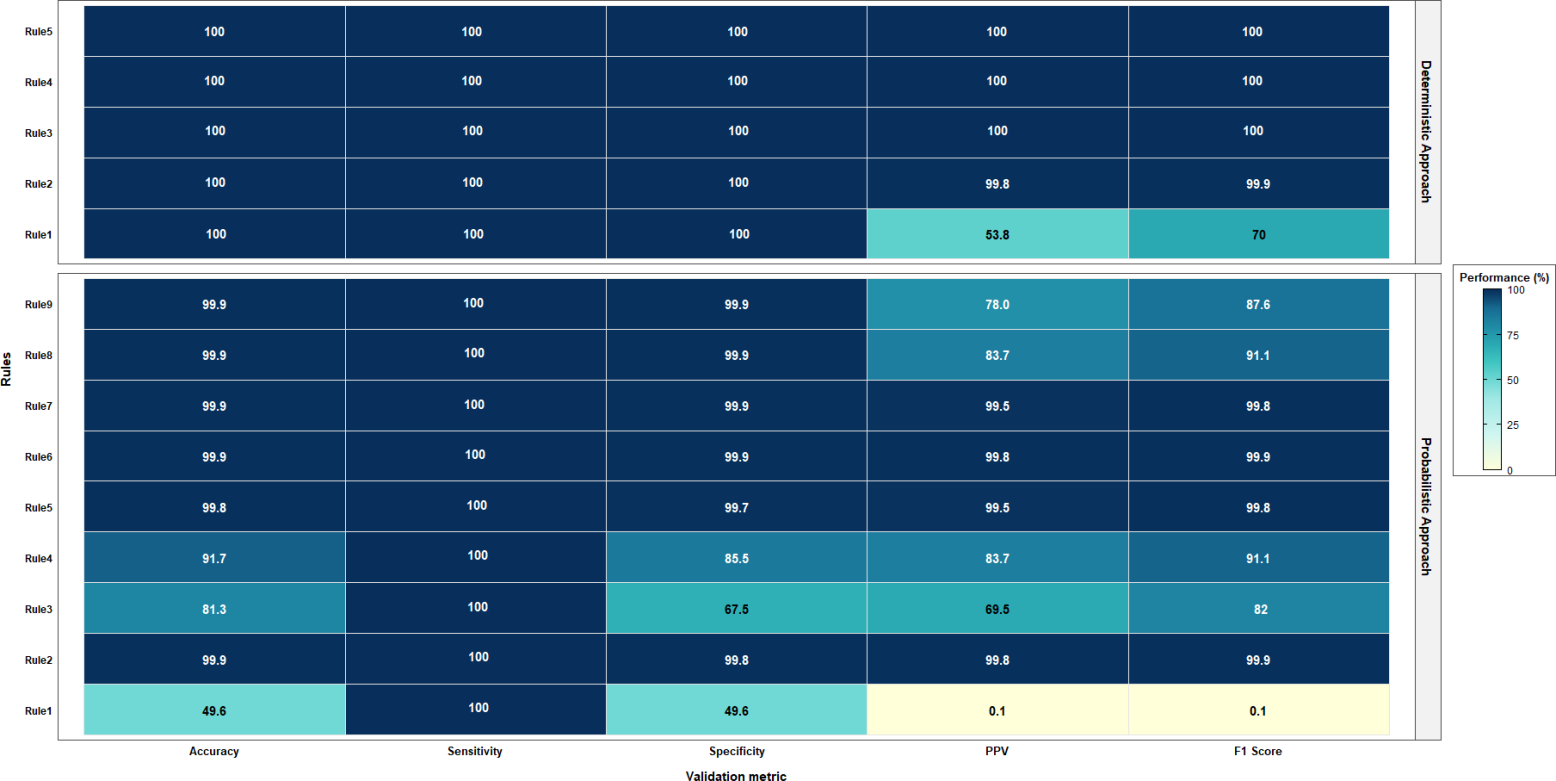
Performance comparison of deterministic and probabilistic approaches. PPV: positive predictive value.

**Figure 2.**
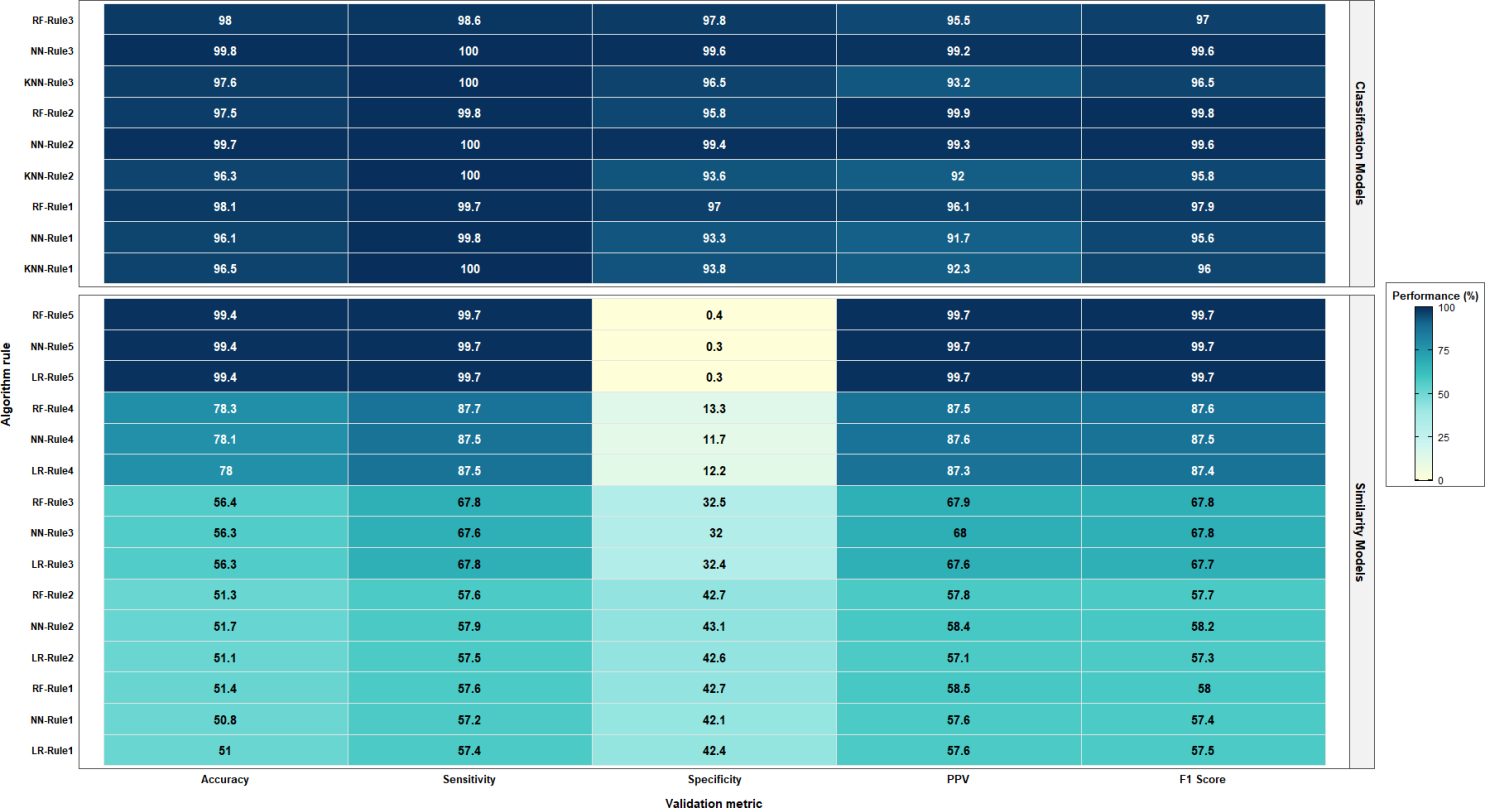
Performance comparison of machine learning models. KNN: K-nearest neighbor; LR: logistic regression; NN: neural network; PPV: positive predictive value; RF: random forest.

Considering the average *F*_1_ score for the different models in each approach, the deterministic approach achieved the highest average (97.2%), followed by the probabilistic approach (93.9%). Noteworthy, the probabilistic approach using one-to-one linkage achieved an average of 100%, and in both probabilistic and ML approaches, an initial blocking step based on exact matching was performed. Examining the criteria without applying any blocking led to poor performance in the probabilistic method and was infeasible for ML. Figure 1 shows a comparison between deterministic and probabilistic approaches, while Figure 2 shows a comparison between similarity score and classification ML approaches.

### Comparison with Prior Work

Despite the application of a straightforward deterministic algorithm requiring exact matching among all variables of linkage rule, our deterministic solution outperformed the probabilistic method, in contrast to findings from previous studies [21,22]. For instance, Gomatam et al [21] used a forward stepwise deterministic method to evaluate variables independently before constructing matching rules. We used a similar approach and observed high specificity and recall, achieving a perfect score, using this method [21]. The probabilistic linkage was implemented similarly to prior research, and we only allowed for exact matches in the deterministic approach. However, unlike previous studies’ findings where probabilistic linkage outperformed deterministic linkage in most scenarios, our results are comparable, with slightly better performance of the deterministic approach when linkage rules (rather than individual variables) were employed [19,21-23]. Implementing a one-to-one linkage method proposed by the reclin package further improved the probabilistic approach to exceed the performance of the deterministic approach when using similar matching rules [20]. Compared with Röchner et al [16], our ML approach exhibited a lower performance than the deterministic approach using a similar combination of rules. Furthermore, even though NN in the classification method achieved a near-perfect score, it required significantly more computational time than the deterministic approach. Nonetheless, we reached a similar finding to Röchner et al [16] in identifying the NN as the highest-performing algorithm in record linkage.

Computational benchmarking was performed on a standard workstation featuring an Intel Core i7 processor, 16 GB of RAM, and 2 GB of GPU (64-bit Windows OS). From a computational perspective, the most efficient method of linkage was the deterministic approach: each run finished in under one second, *F*_1_ scores were between 70% and 100%, and memory requirements were low (<2 GB). The probabilistic approach required 0.1 to 4.88 seconds (*F*_1_ score 82%‐99.9%), while maintaining moderate memory requirements (<8 GB) and achieving excellent accuracy. In contrast, machine-learning-based methodologies were very computationally intensive requiring: 12 to 7467 seconds (∼0.2‐124 min; *F*_1_ score 57.3%‐99.7%, similarity-based models), and 13 to 16,936 seconds (∼0.2‐282 min; *F*_1_ score 95%‐99.8%, classification-based models), both at a maximum memory requirement of 12 to 14 GB. These data demonstrate that the deterministic and probabilistic approaches are ideal for large scale and routine linkage tasks where the computational efficiency of the method is crucial, while ML-based methods, though highly accurate, demand greater resources and are better suited for smaller or high-value analytic linkages where accuracy outweighs runtime considerations.

### Clinical and Regulatory Implications

Beyond demonstrating methodological aspects, this study supports the assertion that EHR linkage of original sources with their standardized mapping has clinical importance of linking the fragmented data of MS into a multi-institutional timeline that supports relapse reconstruction, mapping of treatment trajectories, and assessment of long-term outcomes. Importantly, this linkage addresses the challenges of RWD in MS by enabling the validation of deidentified records and matching the structured components (diagnoses, labs, prescriptions, utilization) with unstructured data (notes by physicians and radiology reports) to improve phenotype definition when ICD codes are ineffectively applied. The linked dataset supports earlier case identification (using utilization surrogates as proxies when standard scales are not available), risk stratification and compliance monitoring across care sites, and provides more frequent and expandable data for pharmacoepidemiologic and safety surveillance that will be more robust than values obtained from trial groups. At the same time, addressing challenges related to the lack of data sufficiency, burdens related to the gaining of patient-reported outcomes data, evolving criteria for diagnosis, and the need for consistent harmonization and resourcing across systems. This linkage produces an analytically reproducible and available dataset for the practical and generalizable studies related to MS and in support of decisions concerning it, changing otherwise unstructured clinical data into actionable knowledge connecting to clinicians, regulators, and health-system leaders.

With the growing availability of large-scale clinical datasets, opportunities to create more comprehensive patient care pathways through data linkage are expanding. The findings of this study suggest that probabilistic linkage of anonymized datasets using indirect identifiers can substantially improve linkage capacity, minimize computational cost, and maintain patient privacy. Probabilistic linkage has demonstrated a greater ability to recover more pairs that may be missed by deterministic methods (1472-2144 vs 1046-1946) due to its flexibility, highlighting its potential as a complementary method to deterministic linkage. Furthermore, to ensure transparency, we examined the contributions of each linkage variable before developing the matching criteria. This approach allowed us to identify the most influential variables for effective linkage. More particularly, the date of birth and first MS diagnosis date achieved *F*_1_ scores of 59.7% and 48.8%, respectively, suggesting strong linkage capabilities with balanced precision and recall. Moreover, incorporating both variables in the matching rules of the deterministic approach resulted in a perfect record linkage across all performance metrics. This improved linkage capability is beneficial for patients with MS by enabling data integration across different providers and supports an even more unified and comprehensive record of each patient’s journey. Such a broad linkage helps researchers and clinicians track the progression of diseases over time, evaluate treatment effects on symptoms, and track long-term health outcomes.

The study findings are significant for regulatory requirements, especially when conducting policymaking and postmarketing surveillance. The low computational costs of the deterministic approach enable the regulators to evaluate its treatment outcomes and specify any safety issues, whereas the flexibility and open-ended retrieval of the probabilistic approach is remarkable, especially in the case of comprehensive data retrieval from anonymized records. Therefore, using both deterministic and probabilistic approaches is significant for creating a comprehensive clinical data source for policymaking, postmarketing assessments, and monitoring of the healthcare industry. Ultimately, a combination of these approaches can lead to excellent patient outcomes regarding safety and treatment accuracy.

### Limitations

Several limitations must be acknowledged in this study. First, our selection of methods was affected by computational constraints, which highlighted the need for advanced optimization and more powerful hardware for ML models. Discrepancies in patient records count between datasets could also affect generalizability, highlighting the importance of complete data capture in future studies. In our linkage of RWD, we standardized both datasets in terms of variable names and value structure, perhaps influencing the performance of algorithms when linking across different data quality in a real-world application. Furthermore, one patient in SRWEN had an inaccurate date of birth, while 4 patients in both data sets had an unknown nationality. Also, 44 patients in MNGHA and 11 patients in SRWEN had an unknown coverage status. Variables such as other diagnoses, admissions (visits), and medications contain null values (no value), but we believe that these nulls refer to the absence of a clinical event rather than missing entries. Lastly, the lack of an identifier limited our ability to validate the results against a reference standard for the real-world linkage section. Consequently, we could not accurately evaluate the retrieval of matched pairs. Therefore, we used the number of matches solely to describe the linkage performance in real-world linkage.

### Conclusion

The findings of this study demonstrate how probabilistic linkage with indirect identifiers can enhance data integration, particularly for MS research and patient care. In real-world situations where we lack unique identifiers, probabilistic linkage proved to be both flexible and efficient as a complement to traditional approaches. These results provide a more holistic overview of patients with MS care and afford researchers access to an inclusive dataset for studying disease progression, treatment outcomes, and monitoring medication safety. In conclusion, applying probabilistic linkage techniques can be expected to facilitate more informed, data-driven clinical and research practices in managing MS.

## Supplementary material

10.2196/79869 Multimedia Appendix 1 Supplementary tables.

## Abbreviations

CDM: common data model
EHR: electronic health record
ICD10: International Classification of Diseases 10th version
LR: logistic regression
ML: machine learning
MNGHA: Ministry of National Guard Health Affairs
MS: multiple sclerosis
NN: neural network
RERN: Real-world Evidence Network
RF: random forest
RWD: Real-world data
