# Supplementary material for: Linking Electronic Health Records for Multiple Sclerosis Research: Comparative Study of Deterministic, Probabilistic, and Machine Learning Linkage Methods
Source: JMIR Med Inform. 2026 Feb 4;14:e79869. doi: 10.2196/79869 (PMC12872214; doi:10.2196/79869)
Supplement: Multimedia Appendix 1 [file medinform-v14-e79869-s001.docx]

| Table S1: variables available in this study datasets depending on the criterion. | | |
| --- | --- | --- |
| Criteria/Database | **MNGHA** | **RERN** |
| Demographic | Patient identification, enterprise patient identification, sex, date of birth, nationality, eligibility, coverage, region, facility name, marital status, date of death | Patient anonymized identification, sex, date of birth, nationality, coverage, region, facility name, date of death, date of registration |
| Diagnosis | Patient identification, enterprise patient identification, diagnosis date, ICD-10 code, diagnosis description, department, facility name, region | Patient anonymized identification, enterprise patient identification, diagnosis date, ICD-10 code, diagnosis description, department, facility name, region |
| Medication | Patient identification, enterprise patient identification, internal order code, order date, order name, frequency, unit of measurement, route, daily number of times, duration, administered quantity, facility name, region | Patient anonymized identification, internal order code, order date, order name, frequency, unit of measurement, route, daily number of times, duration, administered quantity, facility name, region |
| Visits | Patient identification, enterprise patient identification, type of visit, admission date, discharge date, admission department, discharge department, discharge reason (ER/inpatient), length of stay, region, facility name | Patient anonymized identification, type of visit, admission date, discharge date, department name, discharge reason (ER/inpatient), region, facility name |

| Table S2: Criteria definitions for each approach | | |
| --- | --- | --- |
| **Approach** | **Rule Number** | **Rule criteria** |
| **Deterministic approach** | | |
|  | Rule 1 | Exact match DOB and Sex |
|  | Rule 2 | Exact match DOB and MS DT |
|  | Rule 3 | Exact match DOB, MS DT, and admissions |
|  | Rule 4 | Exact match DOB, MS DT, admissions and other diagnoses |
|  | Rule 5 | Exact match DOB, MS DT, admissions, other diagnoses, and medications |
| **Probabilistic approach** | | |
|  | Rule 1 | No grouping; compared on: DOB, MS DT, admissions, other diagnoses, and medications |
|  | Rule 2 | Grouped on: DOB; compared on: MD Date |
|  | Rule 3 | Grouped on: DOB; compared on: other diagnoses |
|  | Rule 4 | Grouped on: DOB; compared on: Admissions |
|  | Rule 5 | Grouped on: DOB; compared on: Meds |
|  | Rule 6 | Grouped on: MS DT; compared on: DOB |
|  | Rule 7 | Grouped on: Meds; compared on: DOB^a^ |
|  | Rule 8 | Grouped on: Admissions; compared on: DOB^a^ |
|  | Rule 9 | Grouped on: Admissions; compared on: MS DT |
| **Machine learning (classification) approach** | | |
|  | Rule 1 | No grouping; predicted the linkage based on DOB, and MS Date |
|  | Rule 2 | Grouped on: DOB; predicted the linkage based on sex, coverage, region, nationality, facility name, and MS Date |
|  | Rule 3 | Grouped on: DOB; predicted the linkage based on MS Date |
|  | Rule 4 | Grouped on: MS Date; predicted the linkage based on DOB, sex, coverage, region, nationality, facility name, admissions, and meds |
| **Machine learning (similarity score) approach** | | |
|  | Rule 1 | No grouping; compared on: DOB, MS DT, admissions, other diagnoses, and medications |
|  | Rule 2 | Grouped on: DOB; predicted the linkage based on sex, coverage, region, nationality, facility name, MS Date, other diagnoses, admissions, and meds |
|  | Rule 3 | Grouped on: DOB; predicted the linkage based on MD DT, admissions, and meds |
|  | Rule 4 | Grouped on: first MS diagnosis date; predict the linkage based on DOB, admissions, and meds |
|  | Rule 5 | Grouped on: medications; predicted the linkage based on DOB, MS DT, and admissions |
|  | Rule 6 | Grouped on: admissions; predicted the linkage based on DOB, MS DT, and meds |
| DOB: date of birth; ML: Machine learning; MS: multiple sclerosis; MS DT: first multiple sclerosis diagnosis.  a Using MS DT instead of DOB had same results. | | |

| Table S3: Performance comparison of deterministic and probabilistic approaches | | | | | | | | | | | | |
| --- | --- | --- | --- | --- | --- | --- | --- | --- | --- | --- | --- | --- |
| Approach | **Rule** ^a^ | **Pairs** | **TP** | **TN** | **FP** | **FN** | **Accuracy** | **Sensitivity (Recall)** | **Specificity** | **Positive Predictive Value (Precision)** | **F1-score** | **Time (seconds)** |
| Deterministic | | | | | | | | | | | | |
|  | Exact match DOB and Sex | 4,912 | 2,642 | 6,930,080 | 2,270 | 0 | 99.96% | 100.00% | 99.96% | 53.79% | 69.95% | 0.17 |
|  | Exact match DOB and MS DT | 2,648 | 2,642 | 6,932,344 | 6 | 0 | 100.00% | 100.00% | 100.00% | 99.77% | 99.89% | 0.83 |
|  | Exact match DOB, MS DT, and admissions | 2,642 | 2,642 | 6,932,350 | 0 | 0 | 100.00% | 100.00% | 100.00% | 100.00% | 100.00% | 0.11 |
|  | Exact match DOB, MS DT, admissions and other diagnoses | 2,642 | 2,642 | 6,932,350 | 0 | 0 | 100.00% | 100.00% | 100.00% | 100.00% | 100.00% | 0.17 |
|  | Exact match DOB, MS DT, admissions, other diagnoses, and medications | 2,642 | 2,642 | 6,932,350 | 0 | 0 | 100.00% | 100.00% | 100.00% | 100.00% | 100.00% | 0.25 |
| Probabilistic | | | | | | | | | | | | |
|  | No grouping; compared on: DOB, MS DT, admissions, other diagnoses, and medications | 6,980,164 | 2,642 | 3,459,108 | 3,518,414 | 0 | 49.59% | 100.00% | 49.57% | 0.07% | 0.14% | 144.98 |
|  | Grouped on: DOB; compared on: MS Date | 6,212 | 2,642 | 3,564 | 6 | 0 | 99.90% | 100.00% | 99.80% | 99.80% | 99.90% | 0.11 |
|  | Grouped on: DOB; compared on: All other diagnosis | 6,212 | 2,642 | 2,408 | 1162 | 0 | 81.30% | 100.00% | 67.50% | 69.50% | 82.00% | 0.06 |
|  | Grouped on: DOB; compared on: Admissions | 6,212 | 2,642 | 3,054 | 516 | 0 | 91.70% | 100.00% | 85.50% | 83.70% | 91.10% | 0.08 |
|  | Grouped on: DOB; compared on: Meds | 6,212 | 2,642 | 3,558 | 12 | 0 | 99.80% | 100.00% | 99.70% | 99.50% | 99.80% | 0.16 |
|  | Grouped on: MS DT; compared on: DOB | 8,184 | 2642 | 5,536 | 6 | 0 | 99.90% | 100.00% | 99.90% | 99.80% | 99.90% | 0.08 |
|  | Grouped on: Meds; compared on: DOB^b^ | 21,222 | 2642 | 18,568 | 12 | 0 | 99.90% | 100.00% | 99.90% | 99.50% | 99.80% | 0.19 |
|  | Grouped on: Admissions; compared on: DOB^b^ | 824,564 | 2642 | 821,406 | 516 | 0 | 99.90% | 100.00% | 99.90% | 83.70% | 91.10% | 4.88 |
|  | Grouped on: Admissions; compared on: MS DT | 824,564 | 2,642 | 821,176 | 746 | 0 | 99.90% | 100.00% | 99.90% | 78.00% | 87.60% | 4.51 |
| DOB: date of birth; FN: False negative; FP: False positive; MS: multiple sclerosis; MS DT: first multiple sclerosis diagnosis; TP: True positive; TN: True Negative.  ^a^ Grouped on refers to performing exact matching based on the specified variables (i.e., blocking) in order to reduce the number of generated pairs before linkage using the approach.  ^b^ Using MS DT instead of DOB had same results. | | | | | | | | | | | | |

| Table S4: a comparison of m-probabilities and u-probabilities across the variables. | | | | | | | | | | |
| --- | --- | --- | --- | --- | --- | --- | --- | --- | --- | --- |
| Variable/Criteria | **No grouping. Compare on: DOB, sex, coverage, region, nationality, facility name, MS Date, diagnoses, admissions, meds.** | | **Grouped on ^a^:  DOB.  Compare on:  sex, coverage, region, nationality, facility name, MS Date, diagnoses, admissions, meds.** | | **Grouped on ^a^: First MS Date.  Compare on:  sex, coverage, region, nationality, facility name, DOB, diagnoses, admissions, meds.** | | **Grouped on*:  Admissions.  Compare on:  DOB, sex, coverage, region, nationality, facility name, MS Date, diagnoses, meds.** | | **Grouped on ^a^:  Meds.  Compare on:  DOB, sex, coverage, region, nationality, facility name, MS Date, diagnoses, admissions.** | |
|  |  |  |  |  |  |  |  |  |  |  |
|  |  |  |  |  |  |  |  |  |  |  |
|  |  |  |  |  |  |  |  |  |  |  |
| Number of pairs | 6,980,164 | | 6,212 | | 8,184 | | 824,564 | | 21,222 | |
| Variable | **M-probability** | **U-probability** | **M-probability** | **U-probability** | **M-probability** | **U-probability** | **M-probability** | **U-probability** | **M-probability** | **U-probability** |
| DOB | 0.004 | 0.003 | - | - | 0.999 | 0.951 | 0.537 | 0.541 | 0.645 | 0.51 |
| Sex | 0.553 | 0.552 | 0.999 | 0.636 | 0.999 | 0.567 | 0.928 | 0.914 | 0.92 | 0.93 |
| Nationality | 0.943 | 0.944 | 0.999 | 0.977 | 0.999 | 0.951 | 0.415 | 0.285 | 0.523 | 0.233 |
| Coverage | 0.346 | 0.301 | 0.999 | 0.357 | 0.999 | 0.341 | 0.009 | 0.004 | 0.287 | 0.005 |
| Facility | 0.999 | 0.000 | 0.999 | 0.016 | 0.999 | 0.005 | 0.999 | 0 | 0.999 | 0 |
| Region | 0.999 | 0.076 | 0.999 | 0.375 | 0.999 | 0.608 | 0.999 | 0.167 | 0.999 | 0.111 |
| First MS Date | 0.017 | 0.010 | 0.999 | 0.442 | - | - | 0.02 | 0.009 | 0.292 | 0.01 |
| Visits | 0.179 | 0.085 | 0.999 | 0.166 | 0.999 | 0.154 | - | - | 0.687 | 0.68 |
| Diagnoses | 0.329 | 0.369 | 0.999 | 0.325 | 0.999 | 0.336 | 0.324 | 0.385 | 0.767 | 0.681 |
| Medications | 0.004 | 0.003 | 0.999 | 0.003 | 0.999 | 0.002 | 0.016 | 0.022 | - | - |
| Matching Probability | 0.369 | | 0.425 | | 0.323 | | 0.5526413 | | 0.4394731 | |
| DOB: date of birth; MS: multiple sclerosis; M-probability (Match probability): Represents the probability that two field values agree given that the records belong to the same entity; PPV: Positive Predictive Value; U-probability (Un-match probability): Represents the probability that two field values agree given that the records belong to different entities.  M- and u-probabilities estimated by the EM-algorithm  ^a^ Grouped on refers to performing exact matching based on the specified variables (i.e., blocking) in order to reduce the number of generated pairs before linkage using the approach. | | | | | | | | | | |

| Table S5: Comparison of machine learning model performance | | | | | | | | |
| --- | --- | --- | --- | --- | --- | --- | --- | --- |
| Approach | **Rule Number** | **Algorithm** | **Accuracy** | **Sensitivity** | **Specificity** | **PPV** | **F1 Score** | **Time (seconds)** |
| Machine Learning Classification | | | | | | | | |
|  | Rule1 | KNN | 96.50% | 100.00% | 93.80% | 92.30% | 96.00% | 491.38 |
|  | Rule1 | RF | 98.13% | 99.70% | 97.00% | 96.10% | 97.90% | 9351.08 |
|  | Rule1 | NN | 96.10% | 99.80% | 93.30% | 91.70% | 95.60% | 551.34 |
|  | Rule2 | KNN | 96.30% | 100.00% | 93.60% | 92.00% | 95.80% | 13.41 |
|  | Rule2 | RF | 97.50% | 99.80% | 95.80% | 99.90% | 99.80% | 85.12 |
|  | Rule2 | NN | 57.40% | 0.00% | 100.00% | 0.00% | 0.00% | 51.7 |
|  | Rule3 | KNN | 97.60% | 100.00% | 96.50% | 93.20% | 96.50% | 1119.16 |
|  | Rule3 | RF | 98.04% | 98.64% | 97.80% | 95.45% | 97.01% | 16936.95 |
|  | Rule3 | NN | 99.80% | 100.00% | 99.60% | 99.20% | 99.60% | 1364.66 |
| Machine Similarity Score | | | | | | | | |
|  | Rule1 | LR | 51.00% | 57.40% | 42.40% | 57.60% | 57.50% | 15.82 |
|  | Rule1 | RF | 51.40% | 57.60% | 42.70% | 58.50% | 58.00% | 17.25 |
|  | Rule1 | NN | 50.80% | 57.20% | 42.10% | 57.60% | 57.40% | 65.87 |
|  | Rule2 | LR | 51.10% | 57.50% | 42.60% | 57.10% | 57.30% | 13.81 |
|  | Rule2 | RF | 51.30% | 57.60% | 42.70% | 57.80% | 57.70% | 12.24 |
|  | Rule2 | NN | 51.70% | 57.90% | 43.10% | 58.40% | 58.20% | 41.4 |
|  | Rule3 | LR | 56.29% | 67.76% | 32.38% | 67.61% | 67.69% | 15.57 |
|  | Rule3 | RF | 56.43% | 67.81% | 32.48% | 67.89% | 67.85% | 15.39 |
|  | Rule3 | NN | 56.26% | 67.61% | 32.04% | 67.98% | 67.79% | 56.33 |
|  | Rule4 | LR | 78.00% | 87.50% | 12.20% | 87.30% | 87.40% | 37.3 |
|  | Rule4 | RF | 78.30% | 87.70% | 13.30% | 87.50% | 87.60% | 38.76 |
|  | Rule4 | NN | 78.10% | 87.50% | 11.70% | 87.60% | 87.50% | 184.68 |
|  | Rule5 | LR | 99.36% | 99.68% | 0.33% | 99.68% | 99.68% | 766.14 |
|  | Rule5 | RF | 99.36% | 99.68% | 0.36% | 99.68% | 99.68% | 6857.45 |
|  | Rule5 | NN | 99.37% | 99.68% | 0.30% | 99.69% | 99.68% | 7467.65 |
| Bootstrap = 100  DOB: date of birth; KNN: K-nearest neighbor; LR: logistic regression; MS: multiple sclerosis; MS DT: first multiple sclerosis diagnosis; Meds: Medications; NN: Neural network; PPV: positive predictive value; RF: random forest. | | | | | | | | |

| Table S6: Real-world linkage: number of matched pairs using optimal models | | | | | |
| --- | --- | --- | --- | --- | --- |
| Deterministic | | **Probabilistic (select_n_to_m)** | | **Machine learning** | |
| Rule | **No. Pairs (%*)** | **Rule** | **No. Pairs (%^a^)** | **Rule** | **No. Pairs (%^a^)** |
| Exact match on DOB and MS DT | 1946 (86.6) | Grouped on: DOB compared on: sex, coverage, region, nationality facility name, MS Date, admissions, meds. | 1993 (88.7) | Prediction type: similarity score (NN) Grouped on: admissions  Predicted the linkage based on: DOB, MS DT, medications | 789 (35.1) |
|  |  |  |  |  |  |
|  |  |  |  |  |  |
| Exact match on DOB, MS DT, and admissions | 1461 (65.0) | Grouped on: First MS Date. Compared on: sex, coverage, region, nationality facility name, DOB, admissions, meds. | 2144 (95.4) |  |  |
|  |  |  |  |  |  |
|  |  |  |  |  |  |
| Exact match on DOB, MS DT, admissions, and medications | 1046 (46.6) | Grouped on: Admissions. Compared on: DOB, sex, coverage, region, nationality facility name, MS Date, visits, meds. | 1985 (88.3) | Prediction type: Classification (NN) Grouped on: MS Date Predicted the linkage based on: DOB, sex, coverage, region, nationality and facility name, admissions, meds | 2014 (89.6) |
|  |  |  |  |  |  |
|  |  |  |  |  |  |
|  |  | Grouped on: Meds Compared on: DOB, sex, coverage, region, nationality facility name, MS Date, admissions. | 1472 (65.5) |  |  |
|  |  |  |  |  |  |
|  |  |  |  |  |  |
| DOB: date of birth; LR: logistic regression; MS: multiple sclerosis; MS DT: first multiple sclerosis diagnosis; Meds: Medications; NN: Neural network; PPV: positive predictive value; RF: random forest.  ^a^ The percentages represent the proportion of matched records to the total original record count of 2,247. | | | | | |
